# Creation of a universal experimental protocol for the investigation of transfer and persistence of trace evidence: Part 2 – Implementation and preliminary data

**DOI:** 10.1016/j.fsisyn.2021.100164

**Published:** 2021-08-30

**Authors:** Hervé Ménard, Christian Cole, Roy Mudie, Joyce K. Klu, Melissa Lawson, Stephanie Green, Stewart Doyle, Emma H. MacNeill, Bethany Hamilton, Kelly Sheridan, Niamh Nic Daéid

**Affiliations:** a Leverhulme Research Centre for Forensic Science, University of Dundee, Nethergate, Dundee, DD1 4HN, UK; b Centre for Forensic Science, Department of Pure and Applied Chemistry, University of Strathclyde, 204 George Street, Glasgow, G1 1XW, UK; c Centre for Forensic Science, Department of Applied Sciences, Faculty of Health & Life Sciences, Northumbria University Newcastle, Ellison Building, Newcastle Upon Tyne, NE1 8ST, UK

**Keywords:** Transfer and persistence, Protocol, Trace evidence, Proxy, Statistical analysis

## Abstract

This is the second paper on the development and implementation of a universal experimental protocol for transfer and persistence of trace evidence. Here, we present the results of five individual researchers who implemented the universal experimental protocol for the first time. Over 2500 images were collected, computationally analysed and statistically compared. The results were shown to be reliable and consistent under all conditions tested and were used to model the rate of loss of transferred particles over a 7-day timescale. The protocol was additionally extended to include a test of camera settings. The protocol was found to be useable and robust in this preliminary trial paving the way for it to be deployed more widely.

## Highlights

• An application of a universal protocol for transfer and persistence for the first time.
• Over 2500 raw images collected from 57 highly replicated transfer experiments and 2 persistence experiments.
• Application of a mathematical model to experimental persistence data.

## 1 Introduction

The presence of trace evidence recovered from individuals or surfaces may provide important information at the intelligence gathering and evidentiary stages of a criminal investigation. However, trace evidence and understanding the weight of evidence that such traces may provide in a given framework of circumstances related to a specific case may not always be used to its best advantage particularly as the transfer and persistence of most materials is largely unknown [1]. As such, there is a need for more empirical and foundational research [2,3] upon which to base the interpretation and evaluation of a recovered trace material.

Transfer and persistence experiments relating to a variety of trace materials such as DNA, soil, pollen, GSR, fibres as well as proxy materials have been published over recent years [[4], [5], [6], [7], [8], [9], [10]] adding to the experiential knowledgebase of practitioners. However, there can be inconsistencies in the findings, for example, there is little agreement in how long GSR persists on a person's hands [10]. Furthermore, although various studies may have investigated similar effects and conditions on similar materials, there has been little commonality in the methodologies used. For example, Bull et al. [9] brushed pollen or sprinkled powder or flicked flint onto swatch materials which were then pinned and worn for up to 24 h to study persistence, and Webb et al. [7] precisely counted pollen grains which were transferred to test material. The comparison of results between experiments which lack a common approach is challenging, requires assumptions to be made and may result in erroneous conclusions. The raw data produced by the studies are often not made available with the publications thus losing a valuable resource which would be of use to future experimentalists. Szkuta et al. [5] did provide all the raw data as supplementary data but this was unusual. Most studies tend to provide summary statistics (mean, standard deviation or linear model parameters) in tables within the papers if the data is present at all. Summary statistics only give a partial view of the data and may not fully represent the variability present within. Results which are not underpinned with the raw data are not as robust as they perhaps should be which is, in part, a reason why the Findable, Accessible, Interoperable, Re-useable (FAIR) [11] guidelines were established in 2016 for the management and stewardship of scientific data [12].

This paper forms the second part of the design, implementation and dissemination of a universal experimental protocol developed to investigate the transfer and persistence of trace materials. Part 1 [13] provided the background and described the experimental protocol in detail and here we present the results of an initial set of five transfer and persistence experiments and assess the analytical workflow with real data. The experiments were undertaken by five students across three institutions allowing for consistency of the implementation of the experimental protocol to also be assessed. We reveal some of the early insights whilst being cognizant that they are not conclusive nor complete. In addition, a demonstration is made of the flexibility of the protocol to incorporate extensions of scope, in this case an exploration of camera settings.

## 2 Materials and methods

### 2.1 Transfer experiment

A set of baseline transfer experiments were performed by five independent researchers as described in the universal experimental protocol presented in Menard et al. [13]. Briefly, small quantities of UV powder mixed with flour in a 1:3 (by weight) mixture [8,9,13] were sprinkled on a 3 cm × 3 cm central area of a 5 cm × 5 cm cotton swatch used as the ‘donor’ material. A second swatch of the ‘receiver’ material, 5 cm × 5 cm was placed on top or the donor material and a weight of known mass placed upon both materials for a specific time. The mass and time combinations are presented in Table 1.

**Table 1:** Grid of the baseline experimental conditions for UV powder transfer combinations. The time and mass combinations for the baseline experiment are shown with a tick mark.

		Contact time (s)			
		30	60	120	240
Mass (g)	200		✓		
	500		✓		
	700		✓		
	1000	✓	✓	✓	✓

Following removal of the weight, the donor and receiver materials were carefully separated and the receiver material retained for further persistence experiments. For each replicated transfer experiment, a total of five images were collected under UV light illumination of the sample;

• Photo No. 1 (P1): Donor material background prior to addition of UV powder
• Photo No.2 (P2): Receiver material background prior to transfer
• Photo No. 3 (P3): Donor material after addition of UV powder
• Photo No. 4 (P4): Donor material post transfer
• Photo No. 5 (P5): Receiver material post transfer

Each transfer experiment for a given mass and time combination was repeated 6 times with fresh swatches used in each case, making of a total of 30 images per mass/time combination. The baseline transfer experiments were completed with cotton as the donor material and wool or nylon as the receiver materials and by differing numbers of the five researchers as presented in Table 2.

**Table 2:** Number of researchers that completed each experimental combination. 4 researchers focused on the transfer protocol only. The baseline experiment for the persistence protocol is 60 s–1000 g combination. One researcher did not submit a complete set of images for the Cotton to nylon 30 s- 1000 g.

Transfer		Contact time (s)			
material	Mass (g)	30	60	120	240
Cotton to wool	200		4		
	500		4		
	700		4		
	1000	4	5	4	4
Cotton to nylon	200		4		
	500		4		
	700		4		
	1000	3	5	4	4

Counting of UV particles was performed computationally via image analysis methods using the open source software package Image J (version 1.52) [14,15] completing the following steps; (1) cropping (if necessary) to the central area of the swatch removing background features; (2) converting colour depth to 8-bit; (3) thresholding the background to remove noise; (4) automatic counting of particles and (5) results stored in a file. An exemplar macro for ImageJ is presented in Table 3 [13]:

**Table 3:** Example of ImageJ macro written for batch processing images.

makeRectangle (498, 462, 2256, 2178);
run (“Crop");
run (“8-bit");
setThreshold (20, 255);
setOption (“BlackBackground”, false);
run (“Convert to Mask");
run (“Analyze Particles … ", “display clear summarize");

**Table 4:** Camera settings.

	Shutter Speed (s)	Aperture (*f* stop)	ISO Setting
C1	1/6	5.6	1600
C2	1/25	2	1600

The transfer ratio is the number of particles which have moved from the donor to the receiver material as a proportion of the total number of particles originally recorded on the donor material prior to transfer. Complete transfer of all particles from the donor to the receiver material would give a transfer ratio of 1 and no transfer of any particles would give a ratio of 0.

The transfer efficiency of a specific activity is the amount of UV powder that has moved to the receiver material related to the amount of UV powder left on the donor material on completion of the transfer event. Transfer efficiency takes into account other factors that can be linked to a specific activity. For example, during the separation of the two textile swatches at the end of the transfer step, some particles may be lost and not counted. This will lead to a transfer efficiency of less than 100%. On the other hand, and in the case of using UV powder as a proxy, some of the particles may be present as ‘clumps’ of powder which may split between the two textiles and are counted on both the donor and the receiver materials. In this case the transfer efficiency will be greater than 100%. The combination of both cases is possible and cannot be individually separated.

The following equations were used to determine the transfer ratio of particles and their transfer efficiency (equations 1, 2, 3, 4)):

1 $$Actual\;Receiver=\;Receiver\;post\;tr\left(Photo\;No.\;5\right)\;-\;Receiver\;bg\left(Photo\;No.\;2\right)$$

2 ActualDonor=Donorpostdep(PhotoNo.3)−Donorbg(PhotoNo.1)

3 $$Transfer\;Ratio=\frac{Actual\;Receiver}{Actual\;Donor}$$

4 $$Transfer\;efficiency=\frac{Actual\;Receiver}{Donor\;post\;dep\left(Photo\;No.\;3\right)\;-\;Donor\;post\;tr\left(Photo\;No.\;4\right)}$$

where, tr = transfer; bg = background; dep = deposition.

For all experiments, the metadata was collected manually and documented in a spreadsheet. Image filenames were renamed manually or with the help of Windows command prompt shortcuts. Files were managed locally by the individual researchers and shared with the LRCFS research group via a Box shared folder. The vast majority of the data was complete and correct although some manual curation was required to ensure all the data and metadata matched up correctly. An LRCFS file renamer application was subsequently developed to aid curation of the completed datasets [13].

A statistical analysis of the effect of camera settings on the particles counted on photos P3, P4 and P5 using either a wool or nylon substrate material. Two camera settings were compared for the six combinations of material and wool using a Mann-Whitney test in R (wilcox.test () function) using a two-sided alternative hypothesis from the null. A Benjamini-Hochberg corrected significance level of 0.05 was set [16].

### 2.2 Persistence experiment

The persistence experiment was an extension of the transfer experiment where the receiver material after transfer had occurred and once the image of the transferred material had been captured, was used as the starting data point, *t*_*0*_, for establishing the persistence of the transferred material given normal wear. To establish a baseline for the persistence of the transferred UV proxy material, a fixed mass and transfer time were selected (1000 g for 60 s). Persistence experiments were conducted where 100% cotton was the donor material and 100% wool and 100% nylon were the receiving materials. The receiver material swatches (n = 6) were attached to outer clothing using four safety pins, one at each corner of the swatch, and the garment was worn by the participant for one week. The receiver material swatch remained uncovered during the experiment and the participant undertook normal indoor (non-sport) daily activity including walking, sitting etc. (Further studies could examine different activity levels involving moderate- and vigorous-intensity physical activities). Images of the fabric swatch were captured under UV light following the baseline transfer experimental protocol outlined in Menard et al. [13] after the following time periods post the transfer event: 30 min, 60 min, 120 min, 180 min, 360 min, 720 min (12 h), 1440 min (24 h), 10080 min (168 h or 7 days) and 40320 min (672 h or 4 weeks). For the final three time periods, the receiver material swatch was worn for periods of at least 8 h in each 12, 24 h and 7 day periods representing normal daily wear. The particle count was determined using ImageJ software as previously discussed until zero counts of particles were reached at which point the specific receiver material swatch was no longer examined. By the end of the experiment, only 2 wool and 3 nylon receiver material swatches (out of 6) had remaining particles after 4 weeks.

The data were fitted using parameters obtained with SSasymp (), a function from the R base package stats [17]. Details of the R-code can be found at https://doi.org/10.5281/zenodo.4772478.

### 2.3 Extension experiment

An extension of the protocol was developed to study the effect of camera settings on the ability to count particles under the UV light used to visualize the swatch of material. A Canon EOS 600D camera with a 50 mm f/1.8 II lens was used and two camera exposure settings were chosen and defined as “C1” or “C2” and this information was added as an additional column in the metadata. The details of the settings are specified in Table 3. A complete baseline experiment (as defined in Table 1) was performed where each image was repetitively captured (n = 6) using two different camera settings C1 and C2.

The image data and count results are available at https://doi.org/10.15132/10000166 and all code for the analysis is available via https://doi.org/10.5281/zenodo.4772478.

## 3 Results

### 3.1 Transfer experiments

**Fig. 1:**
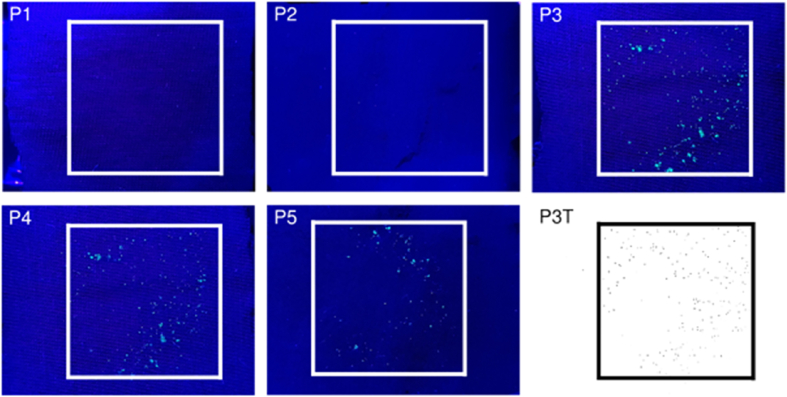
Example images of material swatches during the baseline transfer experiment. Images are labelled by the photo number and comprise a cotton donor material and a Nylon receiver material prior to deposition of the UV powder (P1&P2 respectively); the cotton donor material after deposition of the UV powder (P3); the cotton donor material and Nylon receiver material after transfer (P4&P5 respectively). An example of an image (P3) following ‘thresholding’ in ImageJ is shown (P3T). The square outline represents the approximate location of the 3 × 3 cm central area. The transfer was performed for 60 s and with a 1000 g weight.

Fig. 1 shows exemplar images taken under UV light during the baseline transfer experiment. Any UV fluorescing powder shows up brightly as can be seen in photographs P3–P5 whereas prior to deposition of UV powder no UV fluorescent particles can be seen (photographs P1 and P2). One approach simply described here to determine particle count was to import the images into ImageJ, before applying a threshold to enhance the contrast of the particles against the background and counting the particles using the ‘Analyze Particles’ function. An example image following ‘thresholding’ is also presented in Fig. 1 (panel P3T). Particle counts were collated in a spreadsheet template which included all the relevant experimental parameters together with the source image filename. The spreadsheet metadata was kept together with all the source images for an individual experiment which were then shared for collation with other experiments.

The results presented here were directly taken from the particle count determined by the researchers, using the completed metadata files submitted alongside the respective images. A total of five datasets were collected by the five researchers from two institutions resulting in 2718 images. The results were curated and checked for consistency in terms of files matching their descriptions, no additional analysis of the images was performed beyond what the contributing researchers had done themselves.

**Fig. 2:**
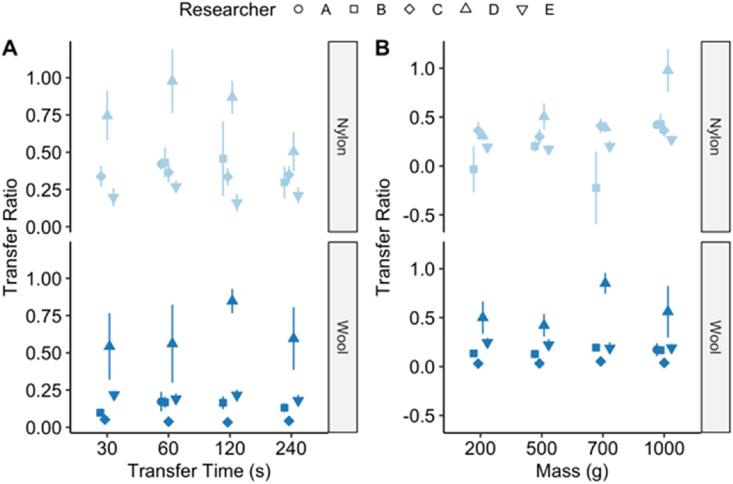
Transfer ratio of UV powder from cotton as the donor material to either wool or nylon as the receiving material over a range of different transfer conditions. Data are shown for five independent researchers (A–E) undertaking the baseline transfer experimental protocol with the recipient materials nylon and wool broken down by transfer time (panel A) or mass (panel B). Data points represent the mean ± std error (n = 6 for most of the individual datasets). Researcher A only collected data for the 60 s and 1000 g with both nylon and wool. Researcher B omitted collecting data for a 30 s transfer time with nylon.

From the raw particle counts obtained from various sets of the replicate images of the five images (P1–P5) taken for each baseline transfer experiment the transfer ratios and transfer efficiencies were determined using equations 1, 2, 3, 4). The summary transfer ratio results are presented in Fig. 2 for nylon and wool. A transfer ratio (calculated from Equation (3)) of zero would show there was no transfer between the donor material and the receiver sample while a value of 1 would indicate there was 100% transfer. The majority of the baseline experiments were repeated 6 times as requested in the protocol, however a small number of experiments had a fewer replicates due to errors or omissions in the submitted data, but this did not detract from the overall trends observed in the data.

Fig. 2 reveals the data broken down as a function of transfer time (panel A) and transfer mass (panel B). The baseline transfer experiments carried out by the five researchers are labelled A through to E.

For both wool and nylon as receiving materials, the transfer time did not seem to have an apparent effect on the transfer ratio for the UV powder. In regard to wool as the receiver material, Fig. 2A bottom, researcher A collected data for the 60 s transfer time only. In the experiments undertaken by researchers B-E the trends observed were flat. The intra-researcher experimental variability was low for all researchers, except for D where the replicate variability dominates.

With nylon as the receiver material, Fig. 2A top, the patterns were similar to wool although the variability between researchers was higher. Researcher A collected data for the 60 s time point only and researcher B did not collect data for the 30 s timepoint. The transfer ratio trends across transfer time were generally flat, except for researcher D, and perhaps researcher B, which suggests a negative relationship between transfer time and transfer ratio. However, the variability in the experiments undertaken by researcher D were high making it difficult to draw concrete conclusions. The experiments undertaken by researcher B were also quite variable whilst the experimental sets generated by the other researchers demonstrated remarkably consistent transfer ratios.

In comparing the two materials in Fig. 2A it was not possible to discern any noticeable differences between the receiving materials in terms of the transfer time. There was some consistency between the data produced by the researchers, however, with D showing high variability and B, C and E controlling the variability better. Despite, researcher A only performing a single time-point, the variability of the experiments undertaken appeared well controlled.

In terms of the mass applied during the baseline transfer experiments, the trends in Fig. 2B for wool and nylon as receiver materials were essentially flat. There was a little apparent difference in transfer when using a 200 g weight vs a 1000 g weight. Again, the data obtained by researcher D demonstrated a greater variability when compared to the other researchers although the agreement across all five when nylon was the receiver material (Fig. 2B top) was generally high. Researcher B demonstrated higher variability at 200 g and 700 g masses as well as a negative transfer ratio, which suggests that the background particle count in either the donor or receiver material was higher than the deposited UV powder. On closer inspection, it was found that one replicate in each of the 200 g and 700 g tests undertaken by this researcher had a donor material with a higher particle count after transfer than before. This can occur when the deposited powder is in clumps which are then broken up during transfer.

**Fig. 3:**
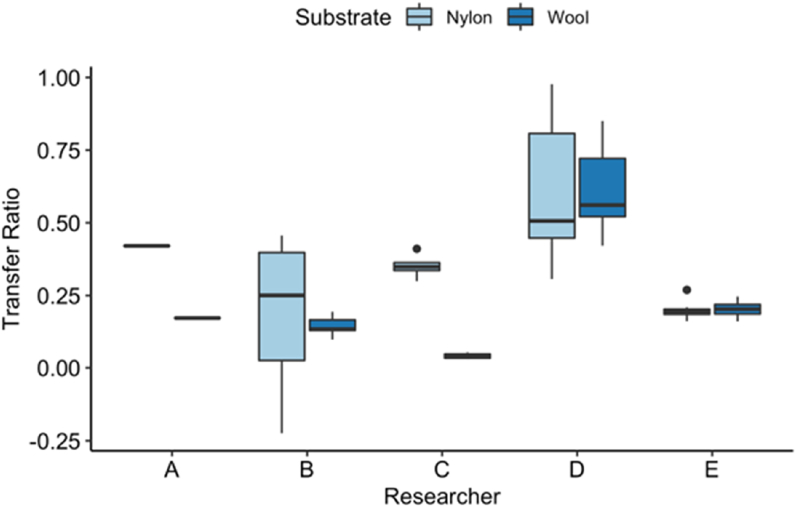
Distribution of transfer ratios revealed by researcher (A–E) categorised by the receiver material (nylon or wool). Boxplots represent the middle 50% of the data in a distribution, the thick horizontal line within the box is the median and whiskers extend from the box by ± 1.5 the inter-quartile range. Points outside the whiskers are considered outliers for being outside the inter-quartile range.

With the effect of both mass and time on the transfer ratio for these initial experiments being minimal it was possible to aggregate all of the data together to determine an overall material effect and to explore whether the different receiver materials had an influence on the transfer ratios. Fig. 3 aggregates all the experimental data for a given receiver material, wool or nylon, and represents the variation within the data set generated by the researchers with boxplots.

Using the median of the data sets as a reference point, the data generated by researchers A, B and C appear to suggest that the transfer ratio was higher with nylon (more particulate material was transferred from the cotton donor material to the nylon receiver material) than wool, although it is worth bearing in mind that researcher A only completed the baseline transfer experiments for one mass and one transfer time combination and the data generated by researcher B much more variable for nylon. For researchers D and E there was no apparent difference between the receiver materials, although researcher D demonstrated a high level of variability in particle counts across all experiments.

Overall, the transfer ratios for either receiver material were relatively low. With the exception of the data from researcher D, the wool transfer ratio medians are all less than 0.25 (25%) and less than 0.5 (50%) for Nylon.

**Fig. 4:**
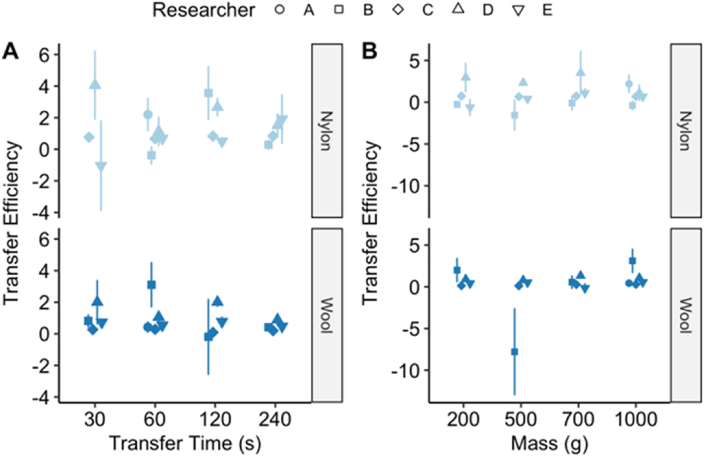
Transfer efficiency of UV powder from cotton as the donor material to either wool or nylon as the receiving material over a range of different transfer conditions. Data are shown for five independent researchers (A–E) undertaking the baseline transfer experimental protocol with the recipient materials nylon and wool broken down by transfer time (panel A) or mass (panel B). Data points represent the mean ± std error (n = 4 for most of the individual datasets with n = 3 and n = 5 on two occasions). Researcher A only collected data for the 60 s and 1000 g with both nylon and wool. Researcher B omitted collecting data for a 30 s transfer time with nylon.

Transfer efficiency is a numerical summary of the process of particle transfer from the donor material to the receiver material whilst attempting to take into account particulate clumps which may disassociate during the transfer process thereby creating apparently more total particles than were originally deposited. Fig. 4 shows the transfer efficiency results for the cotton to nylon and cotton to wool experiments based on the data sets generated by each of the researchers A to E.

A perfectly efficient transfer (transfer efficiency = 1) means that all particles would migrate from the donor to the receiver with no loss nor gain, from de-clumping, in the total number of particles originally deposited on the donor material. A failure to transfer any particles would give a transfer efficiency = 0. The results presented in Fig. 4 reveal a much larger range than 0–1, especially for nylon where the mean transfer efficiencies ranged from −1 to 4.1 when broken down by transfer time (panel A) whereas for wool the mean transfer efficiencies ranged from −0.2 to 3.1. Negative transfer efficiency values indicate that donor particle counts were higher after transfer, whereas values > 1 indicate that the receiver material gained more particles than were on the donor material. Both these effects are likely artefacts caused by clumps of UV powder breaking apart during transfer and highlights that care needs to be taken to avoid clumping of the powder.

### 3.2 Persistence experiments

**Fig. 5:**
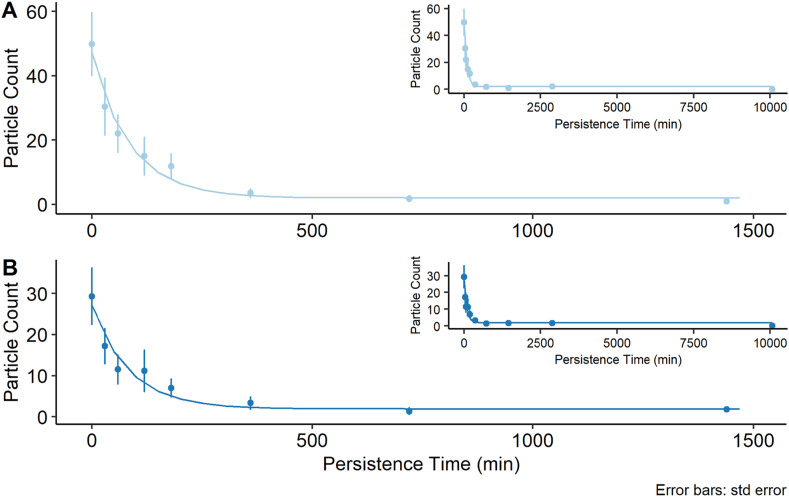
Loss of particles over time during the baseline persistence experiments. Particle counts are shown for transfers performed with a cotton donor material and a 1000 g weight for 60 s to nylon (A) and wool (B) as receiver materials. n = 6 for persistence times 0–720 min, n = 4 for 1440 min, n = 3 for nylon 10080 min and wool 2880 min, n = 2 for nylon 2880 min and wool 10080 min.

The transfer experiment naturally leads to a persistence experiment where the receiver material swatch following transfer then becomes the first time point of a persistence experiment. Fig. 5 shows the progressive loss of particles from two experiments performed with nylon and wool receiver materials following a transfer from cotton as detailed in the Methods and Materials section. The particle counts revealed a loss which has an appearance of a ‘decay’ with the particles completely disappearing by the end of the persistence period of 10,080 min (7 days). An exponential decay has the properties shown in Equation (5),

5 $$y\left(t\right)\;\sim \;y_{f}+\left(y_{0}-\;y_{f}\right)e^{-\alpha t}$$

where *y(t)* represents the loss (or decay) of the number of particles present on the receiving material as a function of persistence time, t. $y_{f}$ is the count where the decay asymptotes, $y_{0}$, is the count at the initial time $t_{0}$ and *α* is the rate constant.

Given these parameters, a non-linear least squares estimate can be fitted to the data once they have been determined. The parameters relating to the fitted curves shown in Fig. 5 generated from the experimental data are detailed in Table 5.

**Table 5:** Parameters of non-linear least squares fit. The R-code can be found at https://doi.org/10.5281/zenodo.4772478. The analysis and results are available in the supplementary information.

Substrate	*Y*_*0*_	*Y*_*f*_	log(*α*)	*α*
Nylon	47.06	2.06	−4.46	0.0116
Wool	27.13	1.91	−4.45	0.0117

The trends observed in Fig. 5 and Table 4 were very similar despite the different starting points. More particles had transferred from the cotton donor material to the nylon receiving material (mean particle count = 49.8) than had occurred between cotton and wool (mean = 29.3), but the rates of loss of the particles once transfer had occurred were almost identical as determined by α. The collection of more data as well as including in the metadata, information such as measurements of the warp and weft, or morphology of the threads within the donor and recipient materials, will increase understanding of which factors affect α most and to what extent. The methodology presented in the Universal experiment [13] provides for such information to also be recorded.

Similar trends in the persistence of trace materials have been seen, using different protocols, with glass [18], pollen, flint particles and UV fluorescent powder [9] giving confidence that the universal baseline experimental protocol and results presented here are representative.

### 3.3 Extension experiments

**Fig. 6:**
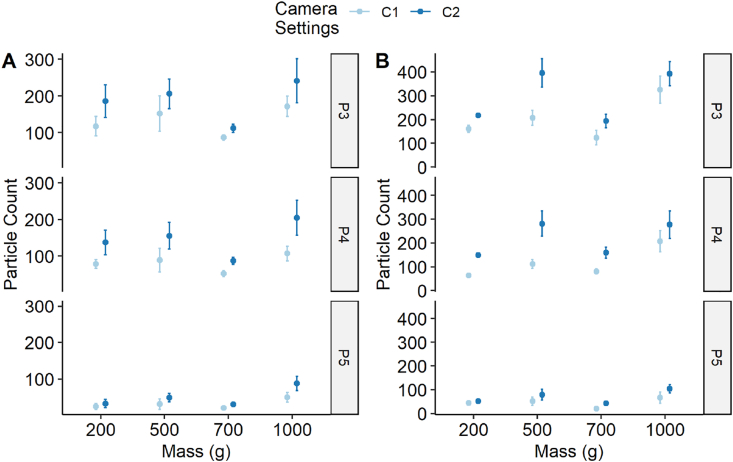
Difference in particle counts for given camera settings. Particle counts are measured for photographs P3, P4 and P5 for different masses used during a 60 s transfer experiment with camera settings C1 and C2 for nylon (A) and wool (B). n = 1 (wool 200 g, C2) – 8 (1000 g, C1) and error bars are standard error of the mean.

In addition to the persistence experiment, a study was performed to determine the effect of different camera settings on the process of counting particles under UV light on the two receiving materials, nylon and wool. Fig. 6 presents the count data as determined for photographs P3, P4 and P5 for nylon and wool for a 60 s transfer experiment over the mass range 200 g–1000 g.

Photographs P1 and P2 are mostly zero counts and critically, represent the background abundance of the target material on the donor and receiver materials (data available at https://doi.org/10.5281/zenodo.4772478). The particle counts for each of the two camera settings, C1 and C2, reveal that there is a systematic difference between the two camera settings with C2 always showing higher counts than C1. The same was observed for the transfer experiments performed over 30 s, 120 s and 240 s timescales (data analysis presented in supplementary information and data available at https://doi.org/10.5281/zenodo.4772478).

**Fig. 7:**
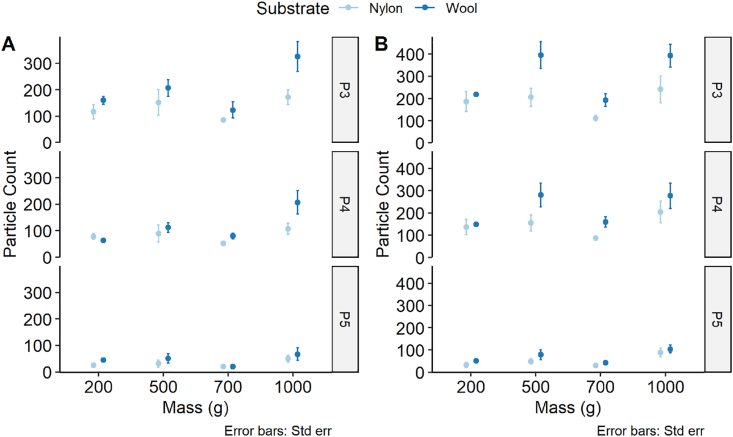
Difference in particle counts for given camera settings and substrate materials. Particle counts are measured for photographs P3, P4 and P5 for different masses used during a 60 s transfer experiment comparing materials with camera settings C1 (A) and C2 (B). n = 1 (wool 200 g, C2) – 8 (1000 g, C1) and error bars are standard error of the mean.

Fig. 7 shows the comparison between the material types for each of the camera settings over the same transfer experimental conditions as presented in Fig. 6. What is notable is that the wool counts are always higher than the nylon counts except for photograph P4, 200 g and C1 where it is lower. This observation taken together with that seen in Fig. 6 suggests C2 is more sensitive at picking up particle counts and that it is also material specific with a stronger effect in wool than nylon. We can test the assumption that the camera settings did not have an effect on the true particle count.

In Figs. 2 and 4 it was revealed that transfer time and mass had a minimal effect so these variables were excluded and all wool and nylon data for each of the three photographs (P3, P4 and P5) were tested using a Mann-Whitney test for a significant effect from the camera settings alone. The data were discrete counts meaning they were assumed to follow a Poisson distribution and the effect of the camera settings on counts was tested. All six combinations of material and photographs were tested for significance and corrected for multiple-hypothesis testing with the Benjamini and Hochberg method [16]. The results are shown in Table 6 for both material substrates.

**Table 6:** Significance statistic of camera exposure setting versus particle counts following Mann-Whitney test.

Substrate	Photo no.	W Statistic	*p*-value	Adjusted *p*-value^a^
Nylon	3	516.0	9.90 × 10^−2^	0.119
Nylon	4	432.0	1.00 × 10^−3^	0.015
Nylon	5	525.5	0.122	0.122
Wool	3	276.0	9.66 × 10^−4^	1.93 × 10^−3^
Wool	4	232.5	1.08 × 10^−4^	6.50 × 10^−4^
Wool	5	265.0	5.71 × 10^−4^	1.71 × 10^−3^

a Raw p-value adjusted for multiple-hypothesis correction using the Benjamini and Hochberg method [16].

**Fig. 8:**
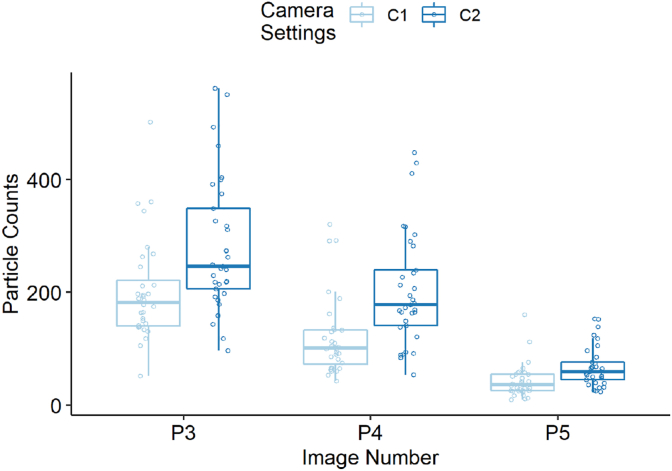
Camera Setting C2 shows higher particle counts with higher variance in Wool. The combined data for all transfer times and masses are collated for wool across photographs P3, P4 and P5. The boxplots show the median count as a dark horizontal line with the box representing the middle 50% of data between quartiles 1 and 3, and the whiskers extend from the box by 1.5 times the interquartile range (IQR).

Four out of the six tests, including all of those where wool was the receiver material, show an adjusted p value of *p* < 0.05. The raw particle counts for all of the photographs P3, P4 and P5 taken of wool as the receiver material for each camera setting are shown in Fig. 8 together with a boxplot summarising the distribution of the data. It is apparent that the median particle counts for C2 are higher than C1 for all three photos, however, it also appears the variance is also higher. This suggests that although the C2 settings were able to identify more particles than C1 they also introduced more noise.

## 4 Discussion

The data and results presented here are an exemplar of how a universal experimental protocol for studying transfer and persistence of physical trace material can be applied to short-term research projects undertaken by different researchers and across different laboratories can be successfully aggregated together for analysis. It is possible to generate relevant data to support the development of a ground truth resource as well as apply the methodology to different research hypotheses for testing. Five sets of experiments undertaken by five researchers are described which have contributed data to the baseline set experimental conditions for the transfer of UV powder to nylon and wool as receiver materials from a cotton donor material. The transfer experiments were augmented with week-long persistence experiments and an extension experiment studying the effect of camera exposure settings.

Combining the data from the five researchers together allows a comparison of the findings to be made. The methodology and data capture are sufficiently detailed to the extent that the combination of the data from the different researchers was straightforward. An important step within the protocol was the automated counting of the UV particles via software and the application of suitable thresholds to differentiate the particles from the background. Unsuitable thresholds can result in over- or under-estimation of the particles present [8], a common issue in image analysis [[19], [20], [21]].

It is as yet too early to draw definitive conclusions, but the data so far collected for the baseline transfer experiment appear to show that the mass and times used do not have a strong effect on the ratio of particles transferred from the donor material to the receiver material (as shown in Fig. 2) nor the transfer efficiency (see Fig. 4). The results hold true for both nylon and wool as the receiving surfaces, despite the slightly different overall transfer ratios (Fig. 3) where they appear to be higher for nylon than wool. The transfer efficiencies are very variable with some being >2.5 or <0, but the majority were in the 0–2.5 range for both nylon and wool. It is possible the more extreme values may be due to clumping/declumping of the UV powder during deposition and transference. More experiments are needed in order to identify what properties of the materials (or other variables) were responsible for this observation. The variability between the experimental data produced by the different researchers was also obvious particularly in the transfer ratio results where researcher D had consistently high variability. Researcher B also had high variability, but less so than D while researchers A, C and E showed well-controlled variability, although A did not include all the combinations of weight and time.

For the two persistence experiments it was encouraging to find that the results returned were very similar to one another even when using different materials. The results also compare well with other studies relating to trace evidence for example GSR studies presented in Blakey et al. [10]. Interestingly, the general trends observed also compare well with fibre transfer and persistence experiments performed by Pounds and Smalldon [22] suggesting a uniform behaviour regarding persistence of traces on fabrics that occurs irrespective of the transferred trace.

It was noted that despite being a requirement, only three of the five researchers fully completed the baseline experimental combinations as defined in Table 1. This was because of a combination of aspects including miscommunication of needs, time limits and missing data during submission. The first and last issues have been subsequently addressed in the universal experimental protocol [13] with more explicit requirements for the baseline experimental process and a more streamlined process for submission of data. The recommendation within the universal experimental protocol was for six replicates to be undertaken for each combination of transfer time and mass as well as for each of the persistence data points. In two cases the data submitted by the researchers did not fulfil this requirement and as such some of the collected data could not be interpreted reliably and therefore were omitted from further analysis. Notwithstanding this, the data that was submitted enabled comparisons to be made between the researchers carrying out the same experimental protocol at different locations and enabled successful outcomes to be reached in regard to the aggregation of the data to reveal common trends.

The data management issues were a significant challenge during the pilot experiments as manually ensuring consistency of filenaming and tracking of metadata required a high level of diligence which took time and effort. Likewise sharing hundreds of files between institutions, although technically straightforward via cloud sharing services, still required considerable manual intervention. As a result two tools were developed, the LRCFS File Renamer and LRCFS Data Uploader web application in order to simplify, verify and streamline the data management and submission process. For more details see our accompanying paper [13].

Through the persistence and extensions sections of this study it can be seen that the protocol was flexible enough to be applied to different scenarios and questions whilst still maintaining the same data format for ease of sharing and analysis. The persistence experiment demonstrated a clear relationship between time since transfer and particle counts for the UV powder and materials under study. After approximately 500 min the majority of the loss of particles had occurred which was similar to other research findings [9,18]. Additional results completed under more conditions will allow further parameterisation of the relationship between time, material, activity type and trace evidence type.

The extension experiment which explored the exposure settings on the camera for capturing particle count data revealed that there may be an influence on the particle count and the camera settings. This emphasises the need for consistency within the experimental protocol being used across the baseline transfer and persistence experiments within an experimental series.

The image processing and data analysis performed using Image J was presented as an example rather than the only way to extract data from the images and subsequent particle count analysis. In making the raw data available, we wish to promote scope for alternative methodologies to also be explored. The emphasis on the provision of the raw data alongside this publication is fundamental to the universal experimental protocol.

## 5 Conclusion

Transfer and persistence of physical trace evidence remains incompletely understood and there is a distinct need to identify the parameters that contribute to how the transfer of material occurs, how efficiently transfer occurs?, how much material may transfer in a given event? and which external factors affect such transfers? Once a material is transferred, how long does the material persist on the receiving surface?, what is the rate of loss? and what aspects of the materials defines those parameters? In collaboration with practitioners, academic discipline leads and others involved in the criminal justice system we have developed a universal experimental protocol for performing transfer and persistence experiments in a consistent, robust and flexible framework. This paper presents a preliminary analysis of the first batch of experiments performed by independent researchers during the prototyping of the protocol. The results demonstrate that data generated from using a UV powder proxy has the potential be used to understand the transfer and persistence phenomenon. The universal experimental protocol is available for wider dissemination and use by the educational, research and practitioner communities to engage with the authors and provide data to grow a “ground truth” dataset on the transfer and persistence of physical materials of value to forensic practice. The protocol is also flexible enough to be extended for use with other more case-relevant particulate trace materials such as gunshot residue [10], pollen [7], glass [18] and many others which will enable the community to work together to generate a dataset which will grow in size and utility into the future.

## Funding

This research was funded by the 10.13039/501100000275Leverhulme Trust RC-1015-011.

## Credit authorship contribution statement

Hervé Ménard: Methodology development, Data Curation, Writing – original draft & editing, Conceptualization. Christian Cole: Methodology development, Formal analysis, Data Curation, Software, Writing – Review & Editing. Roy Mudie: Software, Data Curation. Joyce K. Klu: Formal Analysis. Kelly Sheridan: Methodology development, Supervision. Melissa Lawson, Stephanie Green, Stewart Doyle, Emma MacNeills, Bethany Hamilton: Method development, Investigation. Niamh Nic Daéid: Methodology development, Writing – review & editing, Conceptualization, Funding acquisition.

## Declaration of competing interest

The authors declare that they have no known competing financial interests or personal relationships that could have appeared to influence the work reported in this paper.

## References

[1] Stoney D.A., Stoney P.L. (2015). Critical review of forensic trace evidence analysis and the need for a new approach. Forensic Sci. Int..

[2] Linacre A. (2013). Towards a research culture in the forensic sciences. Aust. J. Forensic Sci..

[3] Mnookin J.L., Cole S.A., Dror I.E., Fisher B.A.J., Houck M.M., Inman K. (2011). The need for a research culture in the forensic sciences. UCLA Law Rev..

[4] Morgan R.M., Allen E., King T., Bull P.A. (2014). The spatial and temporal distribution of pollen in a room: forensic implications. Sci. Justice.

[5] Szkuta B., Ansell R., Boiso L., Connolly E., Kloosterman A.D., Kokshoorn B. (2019). Assessment of the transfer, persistence, prevalence and recovery of DNA traces from clothing: an inter-laboratory study on worn upper garments. Forensic Sci. Int.: Genetics.

[6] Sheridan K.J., Saltupyte E., Palmer R., Gallidabino M.D. (2020). A study on contactless airborne transfer of textile fibres between different garments in small compact semi-enclosed spaces. Forensic Sci. Int..

[7] Webb J.C., Brown H.A., Toms H., Goodenough A.E. (2018). Differential retention of pollen grains on clothing and the effectiveness of laboratory retrieval methods in forensic settings. Forensic Sci. Int..

[8] Levin E.A., Morgan R.M., Griffin L.D., Jones V.J. (2019). A comparison of thresholding methods for forensic reconstruction studies using fluorescent powder proxies for trace materials. J. Forensic Sci..

[9] Bull P.A., Morgan R.M., Sagovsky A., Hughes G.J.A. (2006). The transfer and persistence of trace particulates: experimental studies using clothing fabrics. Sci. Justice - J. Forensic Sci. Soc..

[10] Blakey L.S., Sharples G.P., Chana K., Birkett J.W. (2018). Fate and behavior of gunshot residue—a review. J. Forensic Sci..

[11] Findable, Accessible Interoperable, Re-useable. https://www.go-fair.org/fair-principles/.

[12] Wilkinson M.D., Dumontier M., Aalbersberg I.J., Appleton G., Axton M., Baak A. (2016). The FAIR guiding principles for scientific data management and stewardship. Scientific Data.

[13] Ménard H., Cole C., Gray A., Mudie R., Klu J.K., Nic Daéid N. (2021). Creation of a universal experimental protocol for the investigation of transfer and persistence of trace evidence: Part 1 - from design to implementation for particulate evidence. Forensic Sci. Int.: Synergy.

[14] Schneider C.A., Rasband W.S., Eliceiri K.W. (2012). NIH image to ImageJ: 25 Years of image analysis. Nat. Methods.

[15] Abràmoff M.D., Magalhães P.J., Ram S.J. (2004). Image processing with imageJ. Biophot. Int..

[16] Benjamini Y., Hochberg Y. (1995). Controlling the false discovery rate: a practical and powerful approach to multiple testing. J. Roy. Stat. Soc. B.

[17] R Core Team (2021). http://www.R-project.org/.

[18] Hicks T., Vanina R., Margot P. (1996). Transfer and persistence of glass fragments on garments. Sci. Justice - J. Forensic Sci. Soc..

[19] Doube M., K\losowski MlM., Arganda-Carreras I., Cordelières F.P., Dougherty R.P., Jackson J.S. (2010). BoneJ: free and extensible bone image analysis in ImageJ. Bone.

[20] Guzmán C., Bagga M., Kaur A., Westermarck J., Abankwa D. (2014). ColonyArea: an ImageJ plugin to automatically quantify colony formation in clonogenic assays. PloS One.

[21] Tajima R., Kato Y. (2011). Comparison of threshold algorithms for automatic image processing of rice roots using freeware ImageJ. Field Crop. Res..

[22] Pounds C.A., Smalldon K.W. (1975). The transfer of fibres between clothing materials during simulated contacts and their persistence during wear: Part II—fibre persistence. J. Forensic Sci. Soc..

